# Amphiphilic Block Copolymers: Their Structures, and Self-Assembly to Polymeric Micelles and Polymersomes as Drug Delivery Vehicles

**DOI:** 10.3390/polym14214702

**Published:** 2022-11-03

**Authors:** Ketan Kuperkar, Dhruvi Patel, Leonard Ionut Atanase, Pratap Bahadur

**Affiliations:** 1 Department of Chemistry, Sardar Vallabhbhai National Institute of Technology (SVNIT), Ichchhanath, Surat 395 007, Gujarat, India; 2 Faculty of Medical Dentistry, “Apollonia” University of Iasi, 700511 Iasi, Romania; 3 Academy of Romanian Scientists, 050045 Bucharest, Romania; 4 Department of Chemistry, Veer Narmad South Gujarat University (VNSGU), Surat 395 007, Gujarat, India

**Keywords:** block copolymers, self-assembly, polymer micelle, polymersomes, drug delivery

## Abstract

Self-assembly of amphiphilic block copolymers display a multiplicity of nanoscale periodic patterns proposed as a dominant tool for the ‘bottom-up’ fabrication of nanomaterials with different levels of ordering. The present review article focuses on the recent updates to the self-association of amphiphilic block copolymers in aqueous media into varied core-shell morphologies. We briefly describe the block copolymers, their types, microdomain formation in bulk and micellization in selective solvents. We also discuss the characteristic features of block copolymers nanoaggregates viz., polymer micelles (PMs) and polymersomes. Amphiphilic block copolymers (with a variety of hydrophobic blocks and hydrophilic blocks; often polyethylene oxide) self-assemble in water to micelles/niosomes similar to conventional nonionic surfactants with high drug loading capacity. Double hydrophilic block copolymers (DHBCs) made of neutral block-neutral block or neutral block-charged block can transform one block to become hydrophobic under the influence of a stimulus (physical/chemical/biological), and thus induced amphiphilicity and display self-assembly are discussed. Different kinds of polymer micelles (viz. shell and core-cross-linked, core-shell-corona, schizophrenic, crew cut, Janus) are presented in detail. Updates on polymerization-induced self-assembly (PISA) and crystallization-driven self-assembly (CDSA) are also provided. Polyion complexes (PICs) and polyion complex micelles (PICMs) are discussed. Applications of these block copolymeric micelles and polymersomes as nanocarriers in drug delivery systems are described.

## 1. Introduction

**Figure 1:**
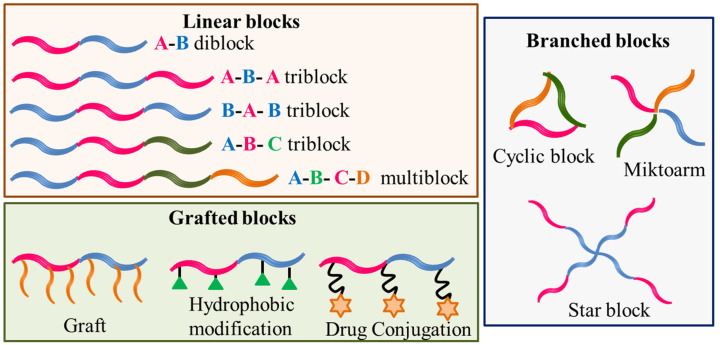
Schematic designer representations of some block copolymer structures.

Amphiphilic block copolymers are constituted of two or more different polymer size blocks, often incompatible, chemically linked in a linear or branched fashion. The blocks can be a neutral polymer (hydrophilic or hydrophobic) or polyelectrolyte (anionic, cationic or zwitterionic) [1,2,3]. A variety of structures can be obtained from such different constituting blocks that are schematically shown in Figure 1.

**Figure 2:**
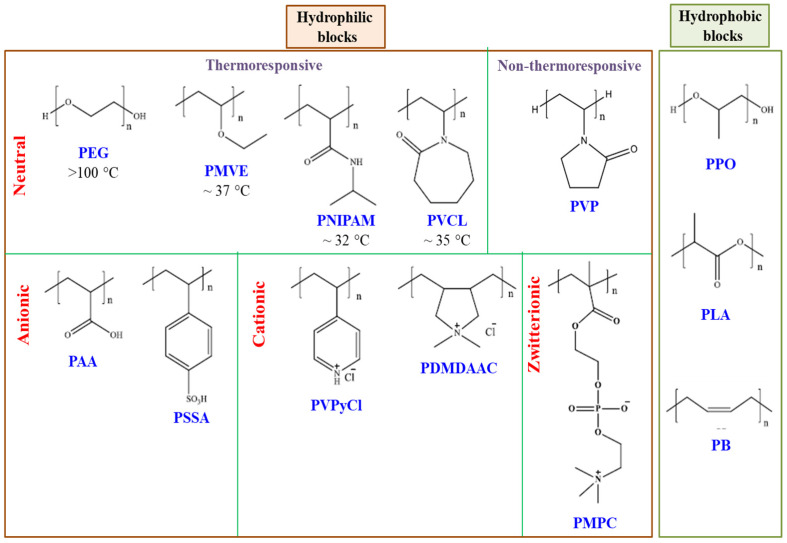
Examples of polymers with varied nature of hydrophilic and hydrophobic blocks.

In most extensively investigated copolymers, diblock and sometimes triblock copolymers have been examined. The hydrophilic block in such copolymers includes polyethylene oxide (PEO) or polyethylene glycol (PEG) condensate type nonionic surfactants (some of which may not be polymeric due to low molecular weight PEO) that are highly useful and commercially available as Tweens^®^, Tritons^®^, Soluplus^®^, Cremophor EL^®^, Solutol HS15^®^, TPGS^®^, etc. Other hydrophilic blocks such as poly(N-isopropylacrylamide) (PNIPAM), polyvinyl caprolactam (PVCL), polyvinyl pyrrolidone (PVP), polyvinyl alcohol (PVA) as well as some polyacids and polybases are also well-known. The hydrophobic blocks can be of polypropylene oxide (PPO), polylactic acid (PAA), polycaprolactone (PCL), polybutylene oxide (PBO), polystyrene oxide (PSO), polybutadiene (PB), polystyrene (PS), polymethylacrylate (PMA), etc. Some common examples of the hydrophilic and hydrophobic blocks considering their charges are illustrated in Figure 2.

Studies have reported the significance of a few Food and Drug Administration (FDA)-approved block polymers such as poly(ɛ-caprolactone) (PCL), polylactic acid (PLA), polyglycolic acid (PGA) and polylactic acid-co-glycolic acid (PLGA) due to their biocompatibility, low cost and biodegradability [4,5,6,7]. A rising research interest in long-term implantable drug delivery systems for a semi-crystalline degradable polymer PLA is mentioned in the reported studies due to its good accelerating rate of bio-absorption, low cost and ease of availability. Additionally, functional polymeric materials with stimuli-responsive blocks have been of interest in recent years [8,9]. External stimuli such as temperature, pH, light, ultrasound, and magnetic field in blocks provide additional properties that modulate the chain conformation in different environments, and can transform the block nature from hydrophilic to hydrophobic and vice versa, thereby resulting in changes of macroscopic characteristics such as solubility and mechanical features that are useful for wide applications in biointerfaces, thin films, coating, and as drug delivery vehicles [10,11,12,13]. These block copolymers develop amphiphilic character and show brilliant surface-activity and micelle formation in aqueous media [1,2]. This behavior is mostly governed by hydrophobic interactions (though in few cases other non-covalent interactions may also operate) in comparison to conventional low molecular weight surfactants [1,2,3]. However, the nature and the molecular composition of blocks and the selectivity of the solvent influences the formation and morphology of nanoaggregates [4,5,6,7].

Like block copolymers, graft copolymers are also surface-active and behave like polymeric surfactants with applications as emulsifiers, surface modifiers, coating agents and compatibilizers for polymer blends. They constitute multiphase polymer systems often containing two or more incompatible polymer chains in which one chain (backbone) has multiple branches on which chains of other polymers are covalently linked through polymerization and are called grafts. Graft copolymers exhibit core-shell self-assembly in selective solvents that can load hydrophobic bioactive substances and hence can be employed as drug delivery vehicles. There exist some interesting review articles that provide updated information on synthesis, identification/analysis, solution behavior and the various application areas of drug delivery [12,13,14,15,16]. Several synthetic and natural polymers can be conveniently transformed into useful amphiphilic graft copolymers.

**Figure 3:**
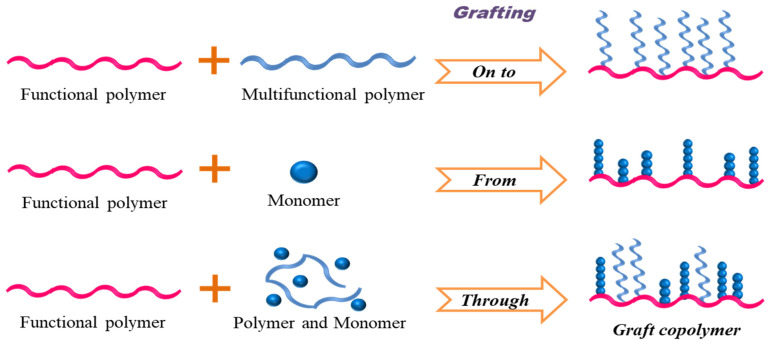
Polymer *“Grafting”* from various methods.

Graft copolymers can be obtained following three common techniques viz “grafting from”, “grafting onto” and “grafting through”. The former method includes the group of anionic sites beside a polymer backbone either by metalation of C–H or C–halogen bonds or by the addition of organometallic compounds such as butyl lithium to reactive vinyl groups. The method provides graft polymers with broad distribution associated with some contamination and degradation. The “grafting onto” approach involves the chain coupling and termination and provides graft copolymers with uniform grafts randomly distributed beside the backbone. Meanwhile, the latter involve macromonomers prepared by living polymerization, reacting with well-defined side chains (Figure 3).

Since the properties of such polymers depend on grafting features, it has now become convenient to finely tune these transformations. The control of grafting/uniformity in the number and size of grafts is a more tedious task in their analysis as compared to their block copolymer counter-parts and so it is difficult to characterize them. However, the grafting of different kinds of monomers to the polymer backbone has long been investigated for enhancing their surface activity, adsorption onto solid surface and micelle formation in solution [14,15,16,17,18,19].

This review is confined to amphiphilic block copolymers which have become important in macromolecular research in the past few decades, and makes an attempt to briefly cover the recent advances in their synthesis strategies, self-assembly behavior, and their emerging scope of application in drug delivery. The self-assembly of block copolymers i.e., in solid state and liquid state leading progressively to varied micellar structures i.e., spherical, ellipsoidal, rod-like, worm-like, polymersome, etc. under different solution conditions is discussed. Possible applications of these polymeric aggregates as drug delivery vehicles are described. The conclusion highlights that the future perspective of this review will attract emerging researchers working on block copolymers to understand their ability to understanding their nanoscale self-assembly which can be used in the fascinating area of polymer research.

Likewise, hydrophobically altered polymers are water-soluble entities which have hydrophobic groups (<2%, mole fraction) in a small amount that directly linked to the main polymer chain, and have recently attracted a lot of attention in oil exploration, paints, mineral separation, cosmetics and pharmaceutical formulations due to interesting synergistic rheological behaviour [5,6,7,8,9,10,11,12]. Several synthetic polymers and polysaccharides have been hydrophobically modified to examine their self-assembly in water [20,21,22,23,24,25,26,27]. Even poorly water soluble drugs have been covalently attached to the polymer backbone to induce amphiphilic character that displays self-assembly in solution. The hydrophobically modified polymers show hierarchical self-assembly in water to form nanoaggregates/nanogels that may be used as potential reservoirs for drug delivery applications [28,29,30,31,32].

## 2. Synthesis Strategies

An ordinarily used polymerization process for unsaturated monomers is free radical polymerisation. However, due to uncontrolled polymerization, the polymers formed are polydisperse with low control on molecular weight. Living anionic polymerization developed in the past 50 years has led to distinct polymers with low polydispersity. However, this suffers from the disadvantage that only few monomers can be polymerised or copolymerized to provide block copolymers with great difficulty. The controlled radical polymerization (CRP) techniques developed in the late 80s could make the synthesis highly convenient and provide block copolymers with well-defined architecture and polydispersity almost equal to one [33,34,35]. The polymerization can be performed in a variety of solvents including water, within a wide temperature range. The CRP techniques use specially designed reagents which act as equilibrium pivot between propagating radicals and dormant species. Typical examples of CRPs are atom transfer radical polymerization (ATRP), reversible addition fragmentation polymerization (RAFT) and nitroxide-mediated polymerization (NMP). ATRP uses a special transition metal catalyst so that the living process can be shut down or restarted depending on the experimental conditions. It turned out to be a robust method for the synthesis of block copolymers with precisely controlled chemical composition and complex architecture using a variety of monomers with low polydispersity [36,37,38]. This technique is very useful in industries involving the polymeric materials for drug delivery applications. The RAFT polymerisation (discovered in CSIRO 1998) is one of the several living or controlled radical polymerization techniques that can be used to produce a pre-chosen polymer with narrow molecular weight distribution with a wide range monomer making use of a chain transfer agent in the form of thiocarbonythio or related compounds [39,40,41]. RAFT agents are capable of polymerizing any monomer that can undergo polymerization by simple free radical polymerization. The nitroxide-mediated polymerization (NMP) makes use of nitroxide initiator to produce polymers of low polydispersity and well controlled stereochemistry [42,43,44,45].

## 3. Self-Assembly

### 3.1. In Solid State

Block copolymers play a significant role in modern macromolecular science by covering the full spectrum of polymer chemistry, polymer physics, and polymer materials [25,46]. Due to their structural features, block copolymers are vastly employed as thermoplastic elastomers in nanopatterning/nanolithography, fuel cells, drug delivery systems, etc. All these applications depend on the nature of monomers that make the blocks, block type (di-, tri-, multi-, star-) and molecular characteristics (total molecular weight and % blocks). Thus, a block copolymer made from the desired polymeric blocks with well-defined molecular characteristics (total molecular weight and % blocks) can be employed for specific applications [3,47].

**Figure 4:**
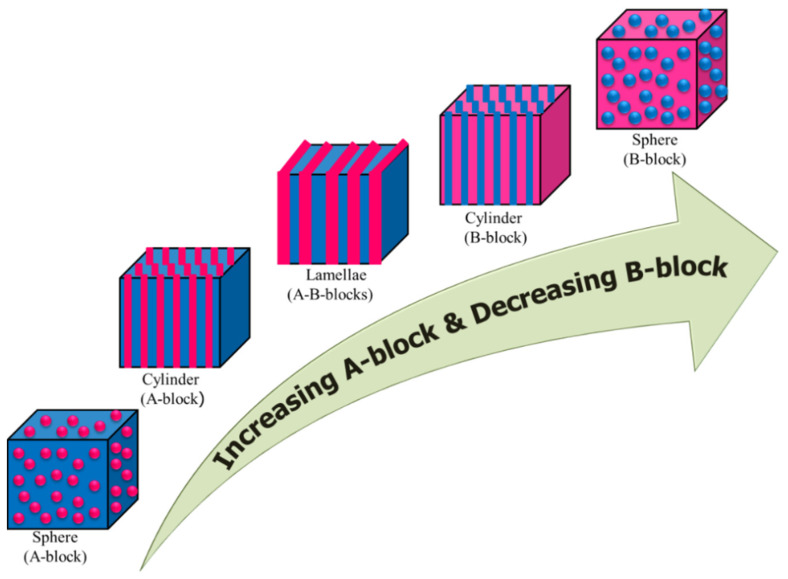
Schematic illustration of diblock copolymer showing various microdomains depending on the relative composition of the blocks.

The presence of incompatible/distinct blocks, their chemical nature, sizes and different structural features make block copolymers excellent multiphase systems that lead to an ironic variety of useful microdomain structures in solid state (in bulk) [16,26,48]. In the case of simple AB diblock copolymers, depending on the volume fraction of the A and B blocks (and to some extent on the chemical nature of the constituting blocks, degree of polymerization (DP) and Flory-Huggins interaction parameter), the microdomain structures that may form are illustrated in Figure 4. The situation becomes more complicated in triblock (ABA and ABC) and multiblock block copolymers.

### 3.2. In Liquid State

**Figure 5:**
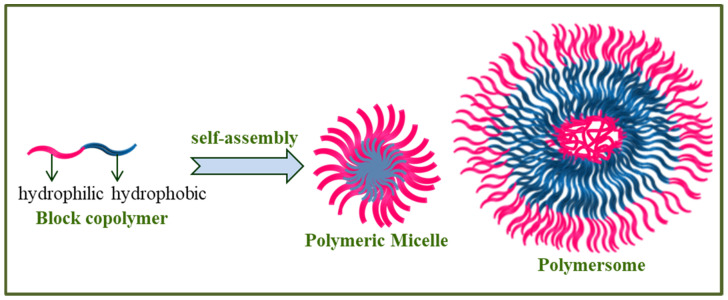
Self-association of amphiphilic block copolymer as polymer micelle or polymersomes in aqueous solution.

Though the research on the block copolymers has been in the forefront of polymer science, interest has exponentially grown and developed due to their distinct solution behavior. In selective solvents (with one block as a good solvent while the other a non-solvent), the amphiphilic block copolymer molecules such as conventional surfactants self-assemble to form nanosized core-shell polymer micelles or bilayered polymersomes in aqueous solution depending on different factors viz., molecular structure of the constituting irreconcilable blocks, the block composition, molecular weight, the hydrophilic and hydrophobic block ratio, temperature, pH, charge, concentration and the presence of additives (electrolytes, non-electrolytes, organic or inorganic compounds, hydrotropes, surfactants), etc. (Figure 5).

Polymer micelles are able to solubilize a large amount of hydrophobic substances. The site of solubilizate depends on the nature of the hydrophobic substance as well as on the two blocks that form core-shell micelles. Polymersomes are capable of solubilizing both the hydrophobic as well as hydrophilic substances. It is for this reason that block copolymers nanoaggregates have become very interesting nanoreserviors for drug delivery systems [49,50,51,52,53].

**Figure 6:**
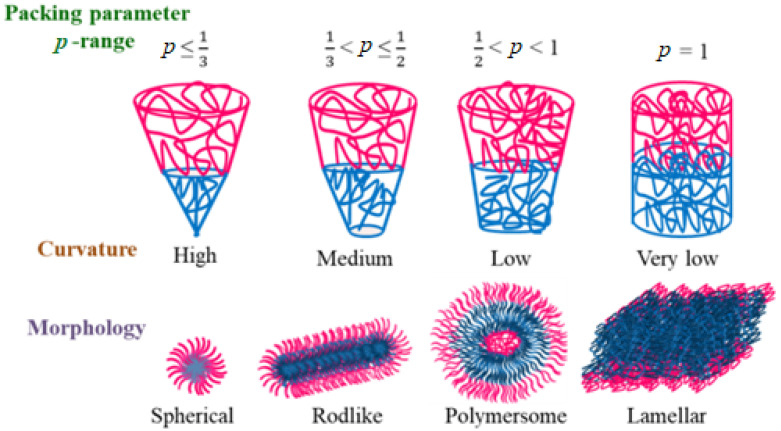
Different block copolymer self-assembly as a function of packing parameter *p*-range.

The formation of nanoaggregates by the self-assembly of amphiphilic copolymers with different morphologies depends on the nature, structure (total mol. wt., %composition of blocks) and solution conditions such as temperature, pH, ionic strength and the presence of additives. Additionally, the interfacial energy between two blocks and their stretching lead to transitions from one morphology to another. For simpler systems, morphological features such as spherical micelles, rod- or worm-like micelles and vesicles can be predicted from the packing parameter *p* = v/a_o_l_c_ (where v is the volume of the hydrophobic segment, a_o_ is the contact area of the head group and l_c_ is the length of the hydrophobic segment). Figure 6 illustrates the micellar transition giving an account of *p* value.

**Figure 7:**
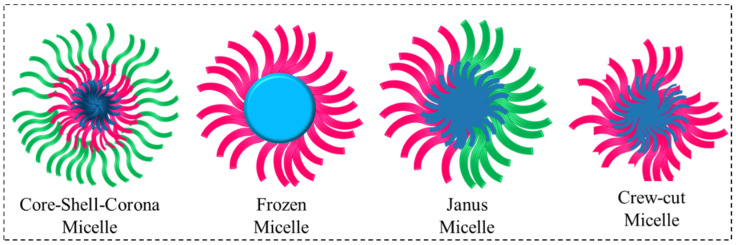
Various form of polymeric micelles from different blocks.

Polymer micelles are kinetically more stable than conventional surfactant micelles [49,50,51,52,53]. Furthermore, depending on the chemical structure of block copolymers, different types of micelles such as frozen micelles, janus micelles, schizophrenic micelles, and crew-cut micelles are formed which are schematically illustrated in Figure 7.

A triblock copolymer with a hydrophobic end block and two hydrophilic oppositely charged polyelectrolyte blocks with the end one of these having high charge density can be seen forming core-shell-corona micelles with ionic corona [54,55]. Amphiphilic block copolymers with hydrophobic block such as polystyrene (PS) and poly(methyl methacrylate) (PMMA) which have high glass transition temperature make the core glassy. These nanoaggregates are stable in solution since molecular motion in the hydrophobic core is arrested and the aggregate has a frozen core. However, these are not equilibrium (dynamic) structures like those from surfactants and block copolymers and do not exchange molecules from micelles [56,57,58,59]. Janus micelles do form as result of self-assembly of ABC type triblock copolymers with the two incompatible and hydrophilic or hydrophobic blocks [60,61,62,63]. The crew-cut core shell micelles are obtained when the core forming block is much larger in size than the hydrophilic block [64,65,66]. Zhang and Eisenberg examined polystyrene-b-poly (acrylic acid) aggregates in water following the dissolution procedure and using good solvent to the solution to induce aggregation of the polystyrene segments and noticed that crew-cut aggregates can show multiple morphologies such as spheres, rods, vesicles, lamellae, large compound micelles and several other structures [66].

**Figure 8:**
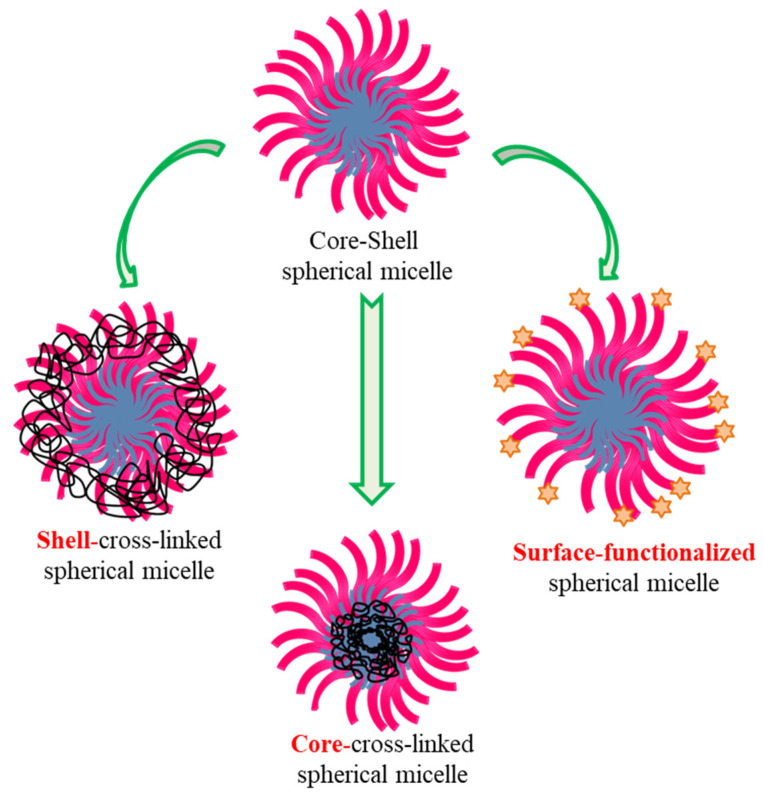
Alteration in the polymeric micelles via cross-linking of core-, shell- and surface-functionalization.

In addition, micelles can be cross-linked as well as surface-functionalized by the attachment of bioactive substances which can be advantageously used (Figure 8). Cross-linked micelles remain undissociated even on extreme dilutions. Additionally, the cross-linking boosts the micelle stability without the drug loading capability fading. It impacts the permeability of the shell and consequently alters the temporal rate of the drug release. Thus, functionalization of BCPs and ease in cross-linking of the core or shell influence the drug loading capacity and release the entrapped hydrophobic drugs [67,68,69,70]. In addition to this, core degraded micelles are also possible where the hydrophobic core degrades and shrinks while the shell is not affected. The amphiphilic copolymers can form mixed micelles with other ionic/nonionic surfactants or polymers to further improve performance. Such strong synergism in mixed surfactant systems can help in fine tuning the features of polymer aggregates for desired applications.

## 4. Types of Micellar Assemblies

### 4.1. Schizophrenic Micelles

**Figure 9:**
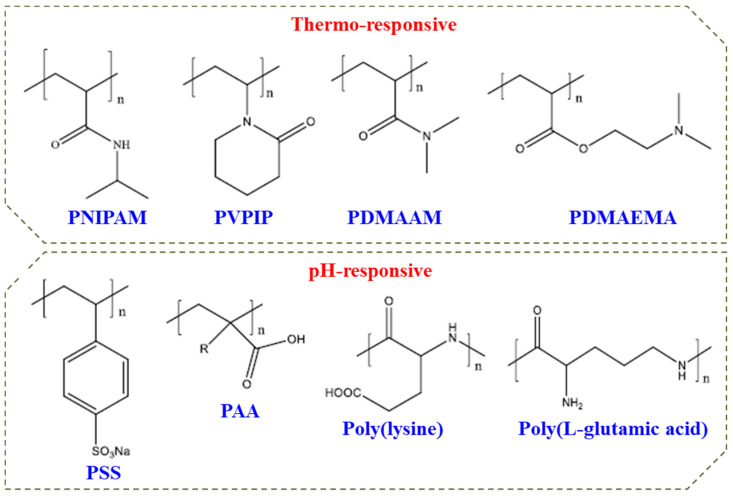
Commonly used polymers in DHBCs.

Double hydrophilic block copolymers (DHBCs) constitute a new class of aqueous multiphase systems and have speedily increased significance with unique behaviour. These can be used for a series of applications in stabilization of colloidal dispersions, crystal growth modification, and polyelectrolyte complexes as drug carrier systems [71,72,73]. Figure 9 shows some commonly used DHBCs which may constitute a responsive block in DHBC.

Most extensively examined stimuli-responsive polymers are thermoresponsive polymers followed by pH-responsive polymers. DHBCs with both such stimuli-responsive blocks can self-assemble to form two distinct micelles with reversed core and shell depending on which of the hydrophilic blocks is turned hydrophobic under the influence of some stimuli such as temperature, solution pH, light, ultrasound, etc. or in the presence of ionic strength or a certain additive. Additionally, micelles can be generated by rendering a polyelectrolyte block hydrophobic through the electrostatic interaction by adding oppositely charged polymers. Studies on DHBC polyionic-thermoresponsive blocks are reported to form micelles with core and shell interchanged under the influence of some stimuli [74,75,76,77,78,79,80,81,82]. For example, PAA-PNIPAM diblock copolymer has shown the micelles with PAA core in very acidic pH at ambient temperature, but under neutral or alkaline pH, micelles with PNIPAM core were formed at a temperature above the LCST of PNIPAM. Additionally, interesting structures can be developed in diblock polyanionic-polycationic copolymers in water and in the presence of salt. A DHBC with a neutral-polyelectrolyte block is molecularly dissolved in water. However, when a solution of an oppositely charged polyelectrolyte is added, self-assembled micelles with complexed ionic core results due to the electrostatic interaction between the oppositely charged blocks. A DHBC with both polyelectrolyte blocks but with opposite charge may not dissolve in water, but undergoes self-assembly in the presence of a small amount of salt. Thus, careful choice of the two hydrophilic blocks and mol. wt./block composition allows the formation of micelles with high efficiency [83,84,85,86].

**Figure 10:**
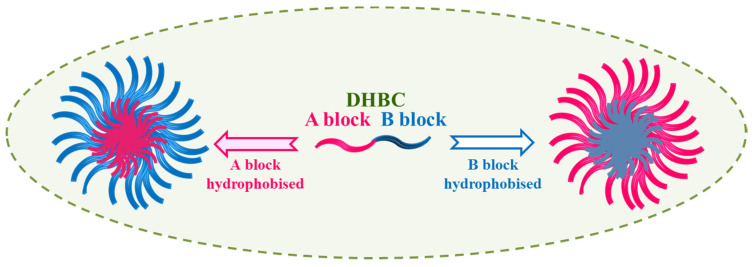
Illustration of Schizophrenic micelles formation.

pH-responsive polymers can release or accept protons by tuning a pH of aqueous solution. These polymers in their assembly involve acid functionality viz. carboxylic or sulfonic acid groups or basic functional groups viz. amines. These so-called “smart and intelligent polymers” are being exploited for a variety of technological and medical applications. The reports on dual responsive as well as multi-responsive polymers undergoing self-assembly do exist [87,88,89,90]. Extensive studies on water-soluble diblock copolymers that reveal so-called “schizophrenic” character have been reported by Prof. Armes and co-workers and few others [91,92]. Schizophrenic micelles (with reversed core and shell) can be formed from a DHBC when each block can turn hydrophobic under some response such as temperature, pH, electronic charge, or solution condition such as concentration, presence of different additives, molecular structure of the constituting irreconcilable blocks, the block composition (molecular weight), the hydrophilic and hydrophobic block ratio, etc. as illustrated in Figure 10.

### 4.2. Polyion Complex Micelles (PICMs)

**Figure 11:**
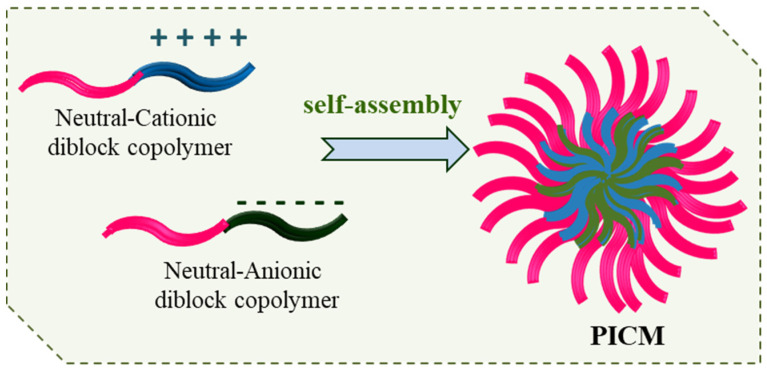
PICMs self-assembly from oppositely charged polyelectrolytes.

Polyelectrolytes (polyacids or polybases) are water-soluble charged polymers that have peculiar aqueous solution behaviour where they dissociate to form a macroion and counterion. The counter ion is condensed onto the macroion or remains as hydrated in bulk solution. The solution behaviour of polyelectrolytes is greatly altered in the presence of salts and also by pH and temperature. Oppositely charged polymers in aqueous solution can form nanoscale water soluble/insoluble complexes or coacervates in solution due to columbic attraction. The formation of soluble/insoluble polyion complexes (PICs) depends on several factors such as the chemical structure, charge, flexibility of the macroions, their mixing ratio concentration, as well as on solution conditions such as pH, temperature, and ionic strength [93,94,95,96]. Polyion complex micelles (PICMs) have an insoluble core of complexed polyelectrolytes and hydrated shell of hydrophilic block when aqueous solution of a neutral-polyelectrolyte block copolymer interacts with an oppositely charged polymer or surfactant (Figure 11).

Fuoss et al. in 1949 first reported the formation of polyelectrolyte complexes (PECs) from the two oppositely charged polymers. However, it was not until 1965 that Michaels et al. observed stable nanosized spherical complexes by mixing two oppositely charged polyelectrolytes by changing mixing composition, presence of salt, and solution conditions such as pH, temperature, presence of salt, etc. Detailed theoretical explanations describing counterion condensation on inter-polyelectrolyte complexes have been provided. Polyelectrolyte complexes (both natural and synthetic) have had great applications in textiles, ink and paper industries as binders, etc. Such PICMs have been of much interest in the last two decades and are good vehicles due to high drug loading in the biomedical field as drug carriers or vectors for gene delivery. Several reviews have been published on their formation, characterization, properties and applications [97,98,99,100].

PICs form predominantly because of electrostatic interaction between the oppositely charged polyelectrolytes though other interactions, such as hydrogen bonding, and hydrophobic interactions may also contribute to complex formation. The gain in entropy due to the release of counter ions from the macroions of the complex formed is dictated by stoichiometric mixing and solution conditions. These can be soluble, stable colloidal dispersions or insoluble coacervates. The effects of ionic strength and pH are remarkable in the formation of PICs from different ones and their characteristics (charged groups, mol. wt., chain flexibility, etc.) and mixing proportion. Low ionic strength allows the complex structure closer to its thermodynamic equilibrium while high ionic strength would shrink it due to the shielding of polyelectrolyte charges.

PICMs form when a polyelectrolyte complex becomes amphiphilic and thus shows self-assembly in analogy to surfactant. Usually, when an amphiphilic block or graft copolymer with one polyelectrolyte moiety in aqueous solution is present, core-shell-charged polymer micelles can form with the core of the hydrophobic block. However, in case of DHBCs (from hydrophilic neutral block-polyelectrolyte block) there is no self-assembly and the solution contains molecularly dissolved polymer. To such a solution, if an oppositely charged polymer is added, with progressive charge neutralization of interacting oppositely charged species, it may so happen that a hydrophobic complex is formed. This may result in the hydrophobic polyelectrolyte complex attached to the hydrophilic chain of the neutral water-soluble polymer chain and gives rise to amphiphilic character and consequently self-assembly. The nanoaggregates formed with complexed core and hydrated shell are polyelectrolyte complex micelles. Several polyelectrolytes, both synthetic and natural, can interact with DHBCs in aqueous mileu form PICM, which can be characterised using spectral, scattering, thermal and microscopic techniques. Interesting structures can be developed on interaction of two DHBCs with oppositely charged polyelectrolyte blocks and the same or a different hydrophilic neutral block. Additionally, these systems can further be designed using stimuli-responsive DHBCs. In short, the PICM (or sometimes PIC polymersomes) that may form can have different morphologies and features that depend on the mol characteristics of DHBCs, polyelectrolytes, their composition in mixed systems and of course on solution conditions [101,102]. There have been some interesting review articles to which readers can refer on PICs and PICM [93,94,95,96,97,98]. We describe some recent studies carried out in the past few years.

### 4.3. Polymerization Induced Self-Assembly (PISA)

**Figure 12:**
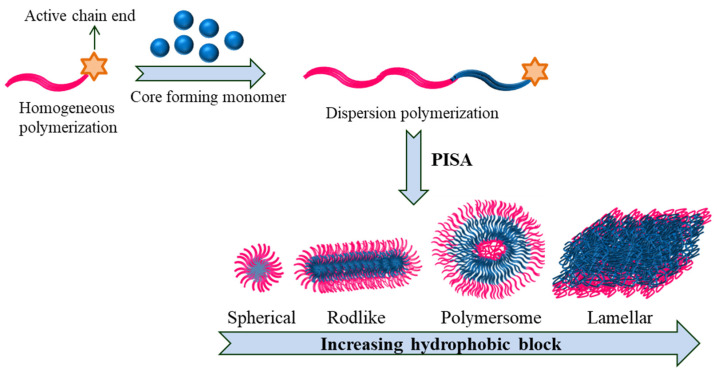
PISA depicting varied shape transition in block copolymers.

Polymerization-induced self-assembly (PISA) is a cost-effective one-pot approach that requires simple procedures to produce polymeric nanoparticles proficiently of various sizes/shapes (sphere, worm-like micelles and vesicles) at high solid concentration as high as 50% (Figure 12).

PISA often involves numerous types of living polymerization techniques; most studies operate RAFT [103,104,105]. Here, a soluble precursor block is chain-extended using a monomer whose corresponding homopolymer is insoluble in the chosen solvent. When the second block reaches a certain level of polymerization, it finally becomes insoluble and results in situ self-assembly and produces nanoaggregates. There have been several articles/ reviews published in the past decade on the production, properties and applications of PISA in the fabrication of a variety of polymeric nanoparticles [106,107,108,109,110].

### 4.4. Crystallization-Driven Self-Assembly (CDSA)

Living crystallization-driven self-assembly (CDSA) has developed as a growth route to form colloidally stable nanoparticles and more complex hierarchical assemblies with the desired size and low size dispersity from crystallizable polymers. The origin of the low dispersity has often been described as initiation being faster than propagation, and termination is absent. When crystal packing forces dominate, a morphological transition is triggered and may lead to elongated nanostructures such as cylinders, nanoribbons, fibres, etc. that depend on crystallization temperature and time, solvent quality, and polymer composition. Thus, the crystallization entails an accurate control of their molecular weight, and their distribution and stereochemistry, and so crystallizable polymers can be used as the immiscible core-forming block to create nanostructures with additional structural characteristics [111,112,113,114,115].

### 4.5. Cross-Linked, Functionalised and Stimuli-Responsive Micelles

Polymer micelles can be cross-linked using different reagents where both the core and shell can be cross-linked. Furthermore, these micelles can be functionalized at the terminal end of the hydrophilic tail (Figure 8). These possibilities provide better opportunity for such micelles to act as nanocarriers. The core cross-linking often increases micelle stability and such cross-linked micelles retain their structure even at low concentration below critical micelle concentration (CMC) of micelle, thereby forming polymeric amphiphile. These micelles can be isolated and re-dissolved as stable nanoparticles and thus prolong the circulating time. The most vulnerable approach is the core cross-linking. This can be achieved using a polymerizable group in the hydrophobic moiety of the block copolymer or adding a polymerizable monomer that stays in the micelle core and then is polymerized using a certain initiator. Sometimes, the decrease in free volume of the micelle core may adversely affect the drug loading capacity. Likewise, the shell of micelles can be cross-linked and the core of the shell cross-linked micelle can disintegrate through degradation/good solvent resulting in nanocontainers as reported by several researchers [67,68,69,70,116]. Another way to alter micelle morphology/characteristics is to functionalize the chain ends of the soluble shells. The chemical functionalization includes the covalent linkage between chain end and an agent that could be ligand. The ligand receptor interaction being highly selective helps in targeting the release of the solubilized drugs to the site of interest. The presence of different additives and salts that can be fine-tuned micellization and micelle characteristics can be also employed for improved solubilization and release. Furthermore, stimuli-responsive block copolymer can form core-shell aggregate which can be used for drug loading. The drug can be released under the influence of external stimuli such as pH, temperature, magnetic response. There are excellent reviews describing cross-linked, functionalised and stimuli- responsive micelles in the context of drug delivery systems [117,118,119,120].

### 4.6. Mixed Micelles

Two or more block copolymers may interact synergistically and form mixed polymeric system with improved features that can be employed as vehicles for drug delivery systems [121,122]. The mixed micelles can have improved physical stability, enhanced solubility and drug bioavailability, and provide better functionality by simple mixing of the constituting amphiphilic copolymers. Polymer mixed micelles from block copolymers as well as polymer-surfactant mixed systems have been of great interest over the past few decades due to their applications in industries and biomedical fields. Several research papers on mixed micelles assembled from block copolymers and their use for drug delivery have appeared in the past and have been critically reviewed. The presence of incompatible blocks drives the block copolymers to a separate phase, but the covalent bond between them prevents phase separation at a macroscopic length scale and occurs at a nanometer length scale, thus producing a rich array of nanostructures in solid state and as well as in selective solvents. These structures exhibit tunable and enhanced mechanical, electrical and chemical properties and thus are significant from a technological view point. Thus, complete information on nanostructures coupled with precise synthesis of block copolymers is highly desired for practical purposes. Additionally, the theoretical studies based on the self-consistent field theory (SCFT) have been used to examine the micro scale phase separation and self-assembly of block copolymers [123,124,125].

### 4.7. Polymer-Drug Conjugates

**Figure 13:**
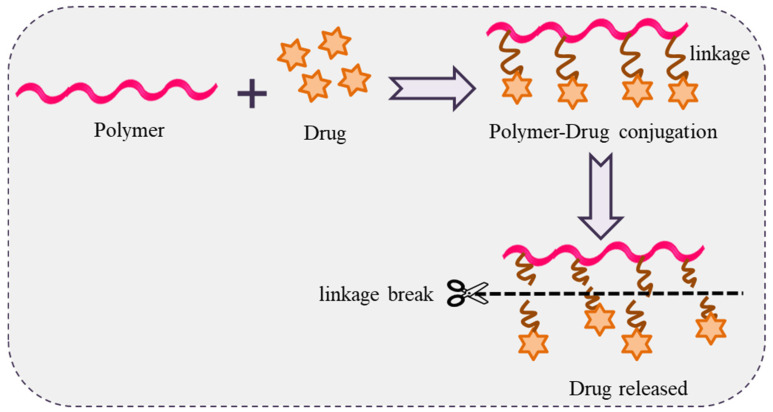
Schematic representation of polymer-drug conjugation with drug release.

Polymer-drug conjugates a contain covalent bond between a water-soluble polymer and a drug. This idea was propounded by Rings Dorf in the mid-1970s. Polymer-drug conjugates behave like a prodrug that resides inactively before cutting of the conjugated bond and release of the active drug (Figure 13). The resulting drug conjugates readily self-assemble in solution and can potentially be used in drug delivery [126,127,128,129,130,131,132].

The conjugate protects the drug, enhances solubility, alters pharmacokinetics, decreases the immunogenicity, and may help develop delivery systems for both active and passive targeting. Several drugs have been conjugated with homopolymers viz. PEG, N-(2-hydroxypropyl) methacrylamide, polyamino acids such as polylysine (PL) and poly L-glutamic acid (PGA), polysaccharides as well as block/graft copolymers as possible drug carriers. Of these, polysaccharides are highly stable, safe, non-toxic, biodegradable, biocompatible and contain several polar/ionic groups that produce bioconjugate when covalently linked with hydrophobic drugs. The polymer-drug conjugation is important in cancer chemotherapy as the clinical use of anticancer drugs suffer from their poor water solubility, short circulation life, non-site-specific targeting, dose-dependent toxicity, and metabolic instability. Instead of novel formulations, careful procedural considerations must be made in their clinical usage since polymer-drug conjugates are evaluated for regulatory reasons as a new chemical entity [126,127,128,129,130,131,132].

## 5. Self-Assembly in Drug Delivery Application

The potential to significantly increase the solubility of poorly water-soluble drugs, lengthen the half-life of the drug in systemic circulation, release the drug at a sustained and controlled rate, deliver the engineered drug in a targeted manner with little interference to healthy cells, suppress drug resistance, and lessen immunogenicity are put forth with the rapid and enormous advances in nanotechnology, and various drug delivery systems [133,134,135]. Biocompatibility and biodegradability along with the high drug loading capacity and controlled release profile are important for the block copolymers acting as vehicles. In contrast to surfactant micelles, polymeric micelles are far superior as these are formed at very low concentration (low CMC), larger in size with low polydispersity, more firm with a slower rate of dissociation, allow retention of loaded drugs for a longer period of time, and achieve higher accumulation at the target site. A cumulative volume of research during the past several years has spurred the development of the polymer-based drug delivery systems [48,102]. Guiraud et al. promoted 25R2 for DNA transfection of skeletal muscle in a similar manner to P105 [136]. Zhang et al. reported the P123 and F127 mixed system for the treatment of multidrug-resistant tumors [137]. Hassanzadeh et al. synthesized F127 and 10R5 mixed micelles for doxorubicin to enhance its therapeutic function and reduce the side effects to normal cells [138]. D. Patel et al. reported the Curcumin solubilization and release profile for polymeric micelles [139,140]. Basak et al. scrutinized the temperature and pH effect on the spherical micelles of F127 which were used for hydrophobic drug encapsulation [141]. Meng et al. reported the Pluronic F-68-coated carbon nanotubes on mesenchymal stem cells [142]. The effectiveness of drug encapsulation and the kinetics of drug release are vital for a block copolymeric micellar cargo in drug delivery. The innovative block copolymer platforms have intrinsic variations. The delivery of drug from drug-loaded micelles in the body depends on their size/shape (typically, an ideal carrier is of uniform size less than about 100 nm) and surface properties. These parameters are strongly dependent on the block constituting polymer chains, their types, molecular weight and block composition along with temperature, pH, and presence of salt in aqueous media. It is therefore not surprising that a variety of copolymers using different monomers and block compositions have been attempted to form micelles or polymersomes that can show favorable drug solubilization with its controlled release profile while the core of polymer micelles is essentially hydrophobic, and can solubilize hydrophobic drugs, and polymersomes with bilayer-like structures (similar to liposomes formed by phospholipids) can solubilize both hydrophobic and hydrophilic drugs. Therefore, the fine tuning of these features is of prime importance to design a good drug delivery vehicle with enhanced stability [143,144,145,146,147,148,149]. Sun et al. synthesized doxorubicin-pluronic F68 conjugate micelles for resistant human erythroleukemic cancer cells [141]. Zang et al. reported an interesting review on P123 micelles used for various kind of drug delivery [142]. Tang et al. described the Porous organic polymers for drug delivery: hierarchical pore structures, variable morphologies, and biological properties [143]. Additionally, the same group reported on poly(l-histidine)-based nanovehicles for controlled drug delivery [144]. Furthermore, the functionalization of the outer surface of these micelles nanoaggregates improves physicochemical and biological properties favorable for receptor-mediated drug delivery. Some polymer-based drug encapsulation and release formulations are currently undergoing Phase I/II clinical trials [145,146,147]. Water-soluble polymers show weak surface-activity and adsorption on solid surfaces while amphiphilic copolymers are surface-active and can stabilize colloidal particles through steric stabilization where the hydrophobic moiety is anchored between particle and hydrophobic chain. The stability can be further improved if the hydrophilic shell is charged and the colloid stability is governed by electrostatic stabilization. Stimuli-responsive block copolymers where the hydrophilic/hydrophobic chains show stimulus response show remarkable adsorption or dispersion stability that responds to the applied stimulus [147,148,149]. In most cases, the hydrophilic segment is PEG, though other water-soluble thermoresponsive blocks from PVME, PVNCL, PNIPAM and others such as PVA, PVP are employed. The hydrophobic moiety can be thermoresponsive PPO (commonly used) or PB, PVP, and biodegradable polymers such as PCL, PLA, and PGLA are used to form core-shell nanoaggregates with high drug loading capacity and exhibiting controlled release. The stimuli-responsive ligand has further widened the possibility for their use in targeted drug delivery.

However, there are some major problems in the clinical testing of some drugs. The in vivo instability and the firm clearance of drug from the blood by the reticuloendothelial system (RES) is one such challenge. To circumvent this problem, nanocarriers with some hydrophilic polymers such as PEG and non-ionic amphiphiles based on it etc. are linked so that highly hydrated flexible PEG successfully escaped from the RES. Additionally, polymer adsorption onto drug-loaded nanoparticle may improve the stability but is undesirable for toxicity issues [150,151]. The possibility of cross-linking the core as well as shell further adds to the advantageous features. Alternatively, an external cross-linker can be used. There are several reports on shell-cross-linkable micelles and those containing functional groups at the chain end of the hydrophilic shell [146,147,148]. Additionally, the graft copolymers of polysaccharides (neutral or charged) backbone with grafts of polymerised hydrophobic chain of monomers can form nanoaggregates suitable for drug delivery [149,150,151].

## 6. Conclusions and Future Perspectives

The present review offers an insight into the self-assembly and structure development of various amphiphilic block copolymers in a targeted drug-delivery system. Self-assembly of amphiphilic block copolymers is spontaneous and often a reversible organization of molecules into nanoaggregates, often called micelles, driven by hydrophobic effect. It is a ubiquitous phenomenon with immense applications in chemical industries, in biology and in biomedical sciences. The advances in controlled polymerization processes to synthesise such amphiphilic block copolymers with a variety of monomers in desired molecular characteristics (structure, mol. wt., block composition) and low polydispersity, and in their characterization techniques have generated much research interest to develop advanced therapeutic systems. During the past several decades, these block copolymers have shown progressive increase in market share, i.e., about 40% of the total polymer production worldwide. However, the market shows dominance of polyethoxylated products, i.e., those with PEG hydrophilic chain as these are often compatible with other types of polymers forming mixed systems with improved solution properties, enhanced performance and commercial importance. As a consequence, the Polymeric micelles (PMs) and polymersomes have become so important due to their potential as nanoreserviors to solubilize hydrophobic therapeutic agents either in aqueous or non-aqueous media in drug delivery systems as powdered or liquid formulations. Such performance is relatively due to more hydrated thermo- and pH-responsive micelles and longer retention period of loaded drugs in polymeric micelles or due to the slower dissociation rate of these micelles. It is therefore important to design block copolymers using monomers (charged/nonionic/stimuli-responsive) to produce a well-defined biocompatible/nontoxic block copolymer that self-assembles into well-characterised nanostructures with high loading capacity for hydrophobic drugs with optimized release kinetic profile. The recent development has shown the rapidly increasing importance of DHBCs and PIC micelles for fruitful future research towards the exploration of new applications. The potential of these micelles is much larger and has excellent properties as pigment stabilizers or crystal growth modifiers. PISA results in in situ self-assembly and produces nanoaggregates which can be used in the fabrication of a variety of polymeric nanoparticles. Furthermore, to enhance the drug loading and release efficacy, grafting of polymers, cross-linking or surface-functionalization of polymers can be achieved. Polymer-drug conjugations help to develop delivery systems for both active and passive targeting. Even the mixed micelles improve the physical stability and enhanced solubility with improved features so that they can be employed as vehicles for drug delivery systems. These nanostructures generated from self-assembly of block copolymers are highly desired platforms for drug delivery applications and are being clinically tested.

The current challenge is to design and formulate the micellar systems that enhance its specific delivery to the site of action with effective penetration ability. Additionally, the drugs transport within the body and its targeted delivery is one of the recent challenges. Overall, in this review, the block copolymer classification, synthesis, various types of block copolymers and its self-assembly with contemporary applications in nanotherapeutics are briefly deliberated. The block copolymers and associated nanostructures grasp massive potential for enhancing the therapeutic efficacy. Despite the exciting growth of block copolymer research in recent years, their utilization in drug delivery systems has not yet been fully exploited. By binding the intrinsic strengths of block copolymers and their synthetic approaches, their potential can be applied to shrink the gap between their design and applications. Such a brief review presented here will surely inspire the readers to infuse the amphiphilic block copolymer features whose mode of action can be modulated as per the desired applications of therapeutics delivery in the field of medicine.
